# Targeting the Ubiquitin Signaling Cascade in Tumor Microenvironment for Cancer Therapy

**DOI:** 10.3390/ijms22020791

**Published:** 2021-01-14

**Authors:** Qi Liu, Bayonle Aminu, Olivia Roscow, Wei Zhang

**Affiliations:** 1 Department of Molecular and Cellular Biology, College of Biological Science, University of Guelph, 50 Stone Rd E, Guelph, ON N1G2W1, Canada; qliu09@uoguelph.ca (Q.L.); baminu@uoguelph.ca (B.A.); oroscow@uoguelph.ca (O.R.); 2 CIFAR Azrieli Global Scholars program, Canadian Institute for Advanced Research, Toronto, ON M5G1M1, Canada

**Keywords:** tumor microenvironment, ubiquitination, E3 ligase, deubiquitinase, immune cells, cancer-associated fibroblasts, extracellular matrix, hypoxia, inhibitors, ubiquitin variants

## Abstract

Tumor microenvironments are composed of a myriad of elements, both cellular (immune cells, cancer-associated fibroblasts, mesenchymal stem cells, etc.) and non-cellular (extracellular matrix, cytokines, growth factors, etc.), which collectively provide a permissive environment enabling tumor progression. In this review, we focused on the regulation of tumor microenvironment through ubiquitination. Ubiquitination is a reversible protein post-translational modification that regulates various key biological processes, whereby ubiquitin is attached to substrates through a catalytic cascade coordinated by multiple enzymes, including E1 ubiquitin-activating enzymes, E2 ubiquitin-conjugating enzymes and E3 ubiquitin ligases. In contrast, ubiquitin can be removed by deubiquitinases in the process of deubiquitination. Here, we discuss the roles of E3 ligases and deubiquitinases as modulators of both cellular and non-cellular components in tumor microenvironment, providing potential therapeutic targets for cancer therapy. Finally, we introduced several emerging technologies that can be utilized to develop effective therapeutic agents for targeting tumor microenvironment.

## 1. Introduction

**Figure 1:**
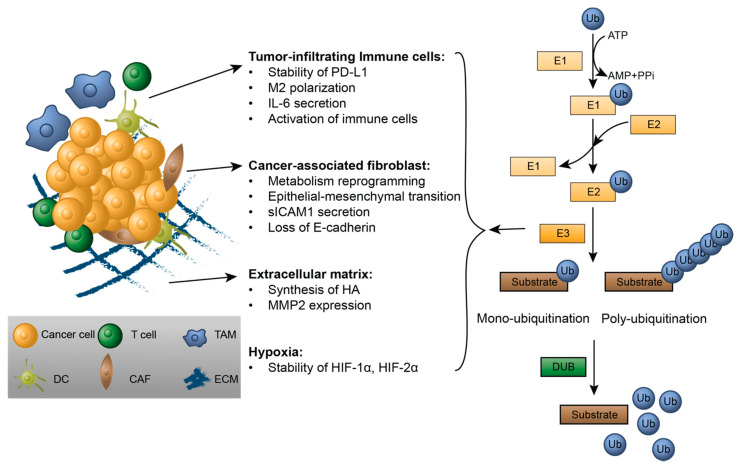
The role of ubiquitin signaling in the tumor microenvironment (TME). Shown on the left, the TME is composed of both cellular and non-cellular components, including tumor-infiltrating immune cells, cancer-associated fibroblasts (CAF) and the extracellular matrix (ECM) and exhibits hypoxia as well. The ubiquitin signaling pathway is shown on the right. Ubiquitin can be attached to substrates by mono-ubiquitination or poly-ubiquitination in the E1-E2-E3 enzymatic cascade and removed from substrates by deubiquitinases (DUB). Multiple E3s and DUBs are implicated in the regulation of multiple TME components, as described in detail in the text.

Ubiquitination is a dynamic and finely regulated post-translational modification pathway that is involved in numerous critical cellular processes, such as selective autophagy [1], the DNA damage response [2] and the innate immune response [3]. This process attaches a small protein called ubiquitin (76 aa, ~8.5 kDa, named after its ubiquitous presence in cells) to substrate proteins through a sequential catalysis pathway involving E1 ubiquitin-activating enzymes, E2 ubiquitin-conjugating enzymes and E3 ubiquitin ligases (Figure 1). The human genome encodes more than 600 different E3 ligases [4], which are categorized to three major families based on different ubiquitin transferring domains: homologous to the E6AP carboxyl terminus (HECT) family, really interesting new gene (RING) family, U-box family and RING-between-RING (RBR) family [5,6]. Conversely, protein ubiquitination is reversed by a group of enzymes called deubiquitinases (DUBs), which catalyze substrate deubiquitination. DUBs are classified into six families based on conserved catalytic domains: ubiquitin-specific proteases (USPs), ubiquitin C-terminal hydrolases (UCHs), ovarian tumor proteases (OTUs), Machado-Joseph disease proteases (MJDs), JAB1/MPR/Mov34 metalloproteases (JAMMs) and motif interacting with ubiquitin-containing novel DUB family (MINDY) [7,8]. DUBs regulate the degradation and activity of their substrates and can serve as both oncogenes and tumor suppressors in cells [9]. Notable ubiquitin modifications include Lys11- and Lys48-linked polyubiquitination, which usually target proteins for proteasomal degradation; and Lys63-linked polyubiquitination, which has non-proteolytic functions and is involved in several signaling pathways [4].

The tumor microenvironment (TME) is the cellular environment where cancer cells reside, which is composed of both cellular components such as various stromal cells, non-cellular components including extracellular matrix and secreted factors, and surrounding blood vessels. Stromal cells, including infiltrating immune cells, cancer-associated fibroblasts, mesenchymal stem cells and endothelial cells, along with cytokines and growth factors, provide a permissive environment for the development of a tumor [10]. The extracellular matrix of the TME provides a scaffold for cancer cells and stromal cells [11]. Key characteristics of the TME include hypoxia [12], high interstitial fluid pressure [13] and acidosis [14]. The TME is closely associated with tumor development and plays critical roles in tumor initiation, progression and metastasis, which makes the TME a prognostic biomarker and potential therapeutic target [14,15]. 

Ubiquitin is an essential post-translational regulator of protein stability and signaling pathways. Not surprisingly, ubiquitination and deubiquitination play critical roles in regulating components of TME through multiple mechanisms and have been investigated extensively. In this review, we summarize how E3 ligases and DUBs in the ubiquitin signaling cascade regulate both cellular and non-cellular components in the TME and how inhibitors targeting TME-related E3 ligases and DUBs contribute to cancer treatment. Finally, we discuss emerging technologies enabling potent and specific modulation of enzymatic activity and how proteasomal degradation of targets can be exploited by engaging E3 ligases.

## 2. Modulating Tumor Microenvironments by Ubiquitination

### 2.1. Tumor-Infiltrating Immune Cells

**Figure 2:**
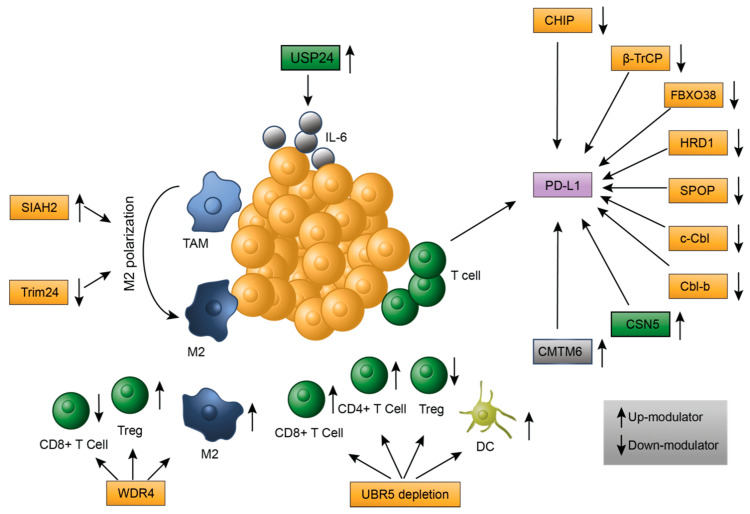
E3 ligases and deubiquitinases (DUBs) involved in modulating immune cells in TME. First, E3s (yellow rectangles), including CHIP, β-TrCP, FBXO38, HRD1, SPOP, c-Cbl and Cbl-b, down-regulate protein levels of programmed death-ligand 1 (PD-L1), and one DUB (green rectangles), CSN5 and another protein, CMTM6 (grey rectangle), can protect PD-L1 from ubiquitination-mediated degradation and stabilize it. Second, the SIAH2 E3 ligase facilitates M2 polarization, whereas the Trim24 E3 impedes this process. Third, the USP24 DUB can promote the transcription of IL-6 and up-regulate its abundance. Finally, WDR4 and depletion of UBR5 can activate and inactivate several immune cells.

Tumor-infiltrating immune cells in the TME include T lymphocytes, B lymphocytes, tumor-associated macrophages (TAM), myeloid suppressive cells (MDSC), mast cells, dendritic cells (DC), neutrophils and natural killer (NK) cells. They can make direct contact with tumor cells, or indirectly via soluble factors, resulting in pro- or anti-tumor effects [15,16]. CD8+ T cells, CD4+ T helper 1 (Th1), M1 macrophages, NK cells and DC have anti-tumor effects, whereas Th2, Th17 and regulatory T cells (Tregs) exhibit both of these opposing effects in animal models and clinical trials; however, B lymphocytes, M2 macrophages, MDSC and mast cells usually have pro-tumor effects [16,17]. The ubiquitin signaling cascade regulates the activity of immune cells and the stability of soluble factors in the TME, resulting in either permissive or suppressive environments for tumor growth (Figure 2). 

Immune evasion is a common feature of tumors in which programmed death-ligand 1 (PD-L1) and its receptor (PD-1) play important roles. PD-L1 is a transmembrane protein that is expressed on the surface of multiple cells, including cancer cells, macrophages, T cells and NK cells, and exhibits elevated levels in various types of tumors. When PD-L1 binds with PD-1, the activity of T cells is inhibited, thereby promoting tumor evasion from immune surveillance. Consequently, PD-L1 or PD-1 inhibition is a promising avenue for developing cancer immunotherapeutic treatments [18,19,20]. The stability of PD-L1 is intricately regulated by various post-translational modifications, including glycosylation, palmitoylation, phosphorylation and ubiquitination [18]. Several E3 ligases have been reported to catalyze the ubiquitination and degradation of PD-L1 in T cells. For example, a U-box E3 ligase CHIP, also known as STUB1, is a well-characterized modulator of cytosolic proteins [5,18] that destabilizes PD-L1 in Tregs through an unknown mechanism [21,22]. The E3 ligase β-transducin repeat-containing protein (β-TrCP) belongs to the Cullin-RING (CRL) E3 ligase, the largest subfamily of RING E3 ligases with more than 200 members, all of which are composed of a Cullin scaffold, a RING-box, an adaptor and a substrate receptor [23]. CRL proteins can be further categorized based on the type of Cullin scaffold. For example, SCF (Skp1-Cul1-F-box) subfamily, which β-TrCP belongs to, is composed of a Cullin 1 scaffold, an Rbx1 RING protein, Skp1 as an adaptor and F-box proteins as substrate receptors [23]. β-TrCP recognizes the typical DSG phospho-degron [18,24] in substrates and catalyzes the phosphorylation-dependent ubiquitination of non-glycosylated PD-L1 in T cells. Glycosylation contributes to stabilization of PD-L1 and interaction with PD-1 [18]. Glycogen synthase kinase 3β (GSK3β) phosphorylates β-catenin and facilitates recognition of PD-L1 by β-TrCP [22,25]. FBXO38 also belongs to the SCF subfamily and catalyzes Lys48-linked polyubiquitination to induce proteasome degradation of PD-1 in tumor infiltrating T cells [26]. The HMG-CoA reductase degradation protein 1 (HRD1), one of the components of the endoplasmic-reticulum associated protein degradation (ERAD) complex, can also function as an E3 ligase that acts upon Ser195 phosphorylated PD-L1 in the process of ERAD [27]. The Speckle-type POZ (pox virus and zinc finger protein) protein (SPOP), an adaptor protein from the CRL E3 family, interacts with PD-L1. Cullin3-SPOP polyubiquitinates and destabilizes PD-L1 and malfunction of SPOP results in a decreased number of tumor-infiltrating cells in mouse models. Intriguingly, the stability of SPOP itself is negatively regulated by Anaphase-Promoting Complex/Cyclosome (APC/C), through interaction with the APC/C adaptor protein Cdh1. SPOP is also positively regulated by the cyclin D/CDK4 complex in a phosphorylation-mediated manner [18,28]. In addition, Casitas B-cell lymphoma (c-Cbl) and Casitas B-lineage lymphoma proto-oncogene-b (Cbl-b) are monomeric RING E3 ligases that negatively regulate PD-L1 expression through inactivating STAT, AKT and ERK signaling in lung cancer [29]. On the other side of the spectrum, DUBs are also implicated in regulating the stability of PD-L1. COP9 signalosome complex subunit 5 (CSN5), a JAMM-family DUB, deubiquitinates and stabilizes PD-L1, thereby escaping T cell-mediated immune surveillance. Expression of CSN5 is induced by NF-κB which is activated by proinflammatory cytokine TNF-α [18,30]. Another protein that stabilizes PD-L1 is the chemokine-like factor (CKLF)-like MARVEL transmembrane domain containing family member 6 (CMTM6). While CMTM6 is not a DUB, it can interact with PD-L1 on tumor cell surfaces and protect PD-L1 from ubiquitination and degradation, which STUB E3 ligase may be involved in, though further studies are needed to delineate the underlying molecular mechanism [18,21]. 

Another important process that is influenced by E3 ligases is tumor-associated macrophage (TAM) polarization. TAM, one of the predominant immune cells in the TME, can be differentiated into two distinct phenotypes when stimulated by different signals. One is the “classically activated” M1, which has anti-tumor effects and usually secretes pro-inflammatory cytokines such as IL-1 (Interleukin-1), IL-12, IL-23 and TNF-α. M1 polarization results from stimulation by Th1 cytokines. The other phenotype is the “alternatively activated” M2, which has opposite effects as M1 and mainly secretes anti-inflammatory cytokines including IL-4, IL-10, prostaglandin 2 (PGE2) and TGF-β upon activation by Th2 cytokines [31,32]. Seven in absentia homologue 2 (SIAH2) is a well-characterized RING E3 ligase in the hypoxia response pathway that induces polyubiquitination-mediated proteasome degradation of nuclear respiratory factor 1 (NRF1) under hypoxic conditions. This results in mitochondrial-dependent metabolic reprogramming with increased levels of the immune factors lactate and PGE2, which facilitates the polarization of TAM to M2 and creates a pro-tumor microenvironment [33]. Tripartite motif-containing protein 24 (Trim24), a RING E3 ligase, catalyzes the ubiquitination of acetyltransferase CREB-binding protein (CBP), which mediates signal transducer and activator of transcription 6 (Stat6) acetylation. Tyrosine phosphorylation of Stat6 activates M2-specific genes, whereas Stat6 acetylation inhibits polarization of TAM to M2. Lys119-linked ubiquitination of CBP by Trim24 promotes the binding of CBP with Stat6, and the increased Stat6 acetylation reduces M2 polarization accordingly. By contrast, *TRIM24* gene expression is suppressed by Stat6 in M2 macrophages [34]. 

Immune cells in the TME can respond to cytokines and secrete their own cytokines as well. IL-6 is a well-known pleiotropic cytokine implicated in numerous physiological pathways and is overexpressed in almost all types of cancers. IL-6 is at high concentration in TME, and can have both pro-inflammatory (through activation of IL-1β and TNF-α in any cell expressing cell receptor gp130) and anti-inflammatory (through inhibition of IL-1β and TNF-α mainly in leukocytes and liver cells) effects in the TME [35]. IL-6 secreted by M2 macrophages and lung cancer cells has pro-tumor effects and its expression is positively regulated by a USP-family DUB, USP24. USP24 deubiquitinates and stabilizes the histone acetyltransferase p300, thereby facilitating histone H3 acetylation of the IL-6 promoter and thus IL-6 transcription. USP24 also inhibits the ubiquitination and subsequent degradation of β-TrCP, which polyubiquitinates IκB and upregulates NF-κB, resulting in increased IL-6 expression. β-TrCP also promotes ubiquitination and degradation of DNA Methyltransferase 1 (DNMT1), a protein that reduces IL-6 promotor methylation and induces its transcription [36]. 

Several E3 ligases are also involved in the activation and deactivation of immune cells. WD repeat 4 (WDR4) is a substrate adaptor in a Cullin4-based CRL E3 ligase that mediates the degradation of promyelocytic leukemia (PML) protein, the gene of which was first described in acute promyelocytic leukemia [37]. PML is a well-characterized pleiotropic tumor suppressor implicated in DNA repair, apoptosis and cancer progression [37] and is usually downregulated in many cancers including lung cancer [38,39]. During the process of WDR4-mediated PML degradation, the CD73 effector protein is responsible for increased amounts of Treg, M2 macrophage and decreased amounts of CD8+ T cells. As a result, it creates an immunosuppressive TME [38,39]. Moreover, ubiquitin protein ligase E3 component n-recognin 5 (UBR5) is an HECT E3 ligase, overexpression of which has been found in multiple cancers [40]. Absence of UBR5 blocks tumor growth partly though mediating immune cells in the TME. In a mouse model carrying the 4T1 breast cancer cell line, UBR5 depletion caused increased CD4+ T cells and CD8+ T cell levels in the spleen and decreased Treg and increased mature DCs in the tumor-draining lymph nodes, therefore inhibiting tumor growth [41]. A recent study [42] by the same group proposed CD8+ T cells were activated via paracrine effects, which may be involved in UBR5’s role in maintaining genome stability and induction of “neoantigens”. However, further studies are needed to delineate the molecular mechanism elicited by UBR5′s E3 ligase activity in regulating immune responses. 

### 2.2. Cancer-Associated Fibroblasts

**Figure 3:**
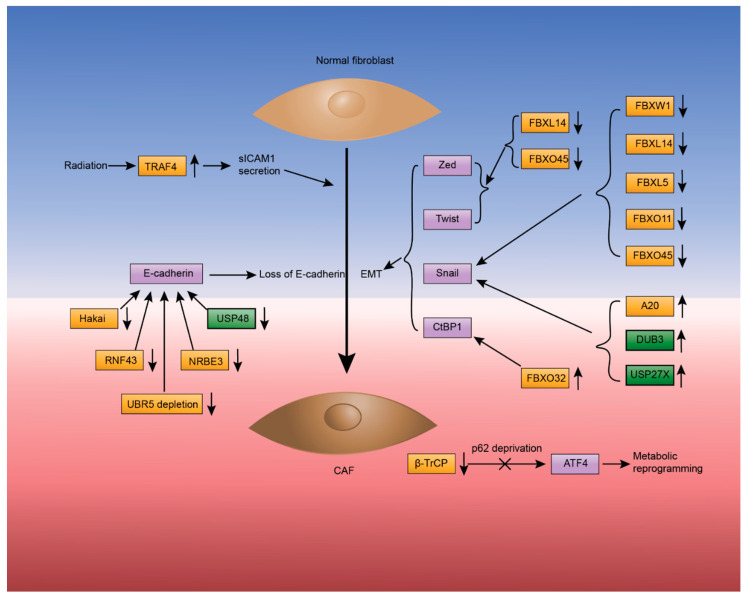
Ubiquitin signaling regulates transformation of normal fibroblasts into cancer-associated fibroblasts (CAF). CAF features in metabolic reprogramming, sICAM1 secretion, epithelial-mesenchymal-transition (EMT) and loss of E-cadherin. For metabolic reprogramming, β-TrCP can down-regulate ATF in a p62 dependent manner as p62 deprivation prevents ubiquitination of ATF and facilitates metabolic reprogramming. TRAF4 E3 up-regulates the secretion of Sicam1. Additionally, the transcription factors Zed, Twist and Snail and their cofactor CtBP1 are involved in EMT. FBXL14 and FBXO45 down-regulate both Zed and Twist. FBXW1, FBXL14, FBXL5, FBXO11 and FBXO45 down-regulate Snail, whereas A20, DUB3 and USP27X stabilize and up-regulate it. FBXO32 up-regulates the cofactor CtBP1. Finally, depletion of a DUB USP48 and several E3 ligases, such as Hakai, RNF43, NRBE3 and UBR5, are implicated in the loss of E-cadherin.

Cancer-associated fibroblasts (CAFs) are one of the major types of stromal cells in the TME. Most CAFs originate from normal fibroblasts activated by cytokines or growth factors. Moreover, mesenchymal stem cells, resident endothelial and epithelial cells also contribute to CAF formation by undergoing endothelial-mesenchymal-transition (EndMT) and epithelial-mesenchymal-transition (EMT) [15]. CAFs play important roles in tumor progression, metastasis and angiogenesis through secreting multiple growth factors, cytokines and chemokines [15,16,43]. CAFs also secrete the matrix metalloproteinases (MMP) such as MMP1, MMP2 and MMP9, which are an important component of the extracellular matrix [10,15]. As described below, several E3 ligases and DUBs have been implicated in reprogramming metabolic pathways, secretion of soluble factors and the EMT (Figure 3).

β-TrCP not only affects protein levels of PD-L1 and IL-6 expression in immune cells, but also the metabolic reprogramming of CAF by catalyzing polyubiquitination of activating transcription factor 4 (ATF4). ATF4 is overexpressed in many types of cancers and regulates the expression of adaptive genes responsible for reprogramming the metabolic pathways that enable cancer cells and stromal cells to tolerate various stresses, including hypoxia, glucose and amino acid deprivation [44]. Glutamine (Gln) serves as a nitrogen source in numerous biosynthetic pathways, deprivation of which is common in cancer cells and stromal cells. Under Gln deprivation conditions, the loss of p62, an autophagy substrate and signaling adaptor, promotes the survival of CAF by blocking β-TrCP’s Lys48 polyubiquitination of ATF, consequently upregulating the protein levels of ATF [45]. The increased level of ATF thereby leads to metabolic reprogramming, which promotes the use of asparagine as a nitrogen source and facilitate EMT.

Tumor necrosis factor receptor-associated factor 4 (TRAF4), an RING E3 ligase, promotes secretion of soluble intercellular adhesion molecule 1 (sICAM1) in normal fibroblasts, which induces angiogenesis and tumor progression [46]. In normal lung fibroblasts, radiation induces Lys63-linked polyubiquitination and upregulation of TRAF4 in a RING domain-dependent manner, which then interacts with and stabilizes the NADPH oxidase (NOX) complexes and ultimately increases endosomal reactive oxygen species (ROS) through a NF-κB mediated pathway. The stabilized NOX complexes subsequently result in increased ICAM1 expression and secretion of sICAM1, which contribute to resistance to radiation, transition to CAF and tumor progression in mouse models [46,47]. 

EMT is a fundamental event for both cancerous and normal cells, and can be triggered by environmental stressors, such as ROS and hypoxia, and extracellular factors, such as TGF-β. Snails are key transcriptional factors that control transcription of EMT-related genes. Despite being stable in cancer cells and activated fibroblasts, Snail1 is unstable and short-lived in normal cells as it is targeted for degradation by multiple E3 ligases. Several well-characterized SCF E3 ligases containing F-box proteins, including FBXW1 (also known as β-TrCP1), FBXL14, FBXL5, FBXO11 and FBXO45, are implicated in the ubiquitination and degradation of Snail1 or Snail2 [48]. A20, also known as tumor necrosis factor α-induced protein 3 (TNFAIP3), has both DUB and E3 ligase activity. A20’s N-terminal domain enables it to function as an OTU DUB, whereas its C-terminal domain enables it to act as an E3 ligase [49]. A20’s E3 ligase activity monoubiquitinates Snail1 at multiple sites and stabilizes Snail1 in the EMT process. Monoubiquitination of three conserved sites on Snail1, Lys206, Lys234 and Lys235, is critical for metastasis in basal-like breast cancers [50]. In addition, DUB3 regulates the polyubiquitination and stabilization of Snail1 in breast cancer. DUB3-mediated Snail1 stabilization is induced and activated by cytokine IL-6 [51] in a CDK4/6-dependent manner [52]. USP27X can also deubiquitinate and stabilize Snail1 when induced by TGF-β and therefore promotes EMT and tumor metastasis [53]. In addition to Snails, Zed and Twist proteins are also important transcriptional factors for EMT. Several F-box proteins, such as FBXL14 and FBXO45, control ubiquitination-dependent degradation of Zed and Twist proteins as well as for Snail proteins [48]. When modulating the transcription of EMT-related genes, Snail1, Zed and Twist proteins usually cooperate with the cofactor C-Terminal Binding Protein 1 (CtBP1). The E3 ligase FBXO32 is implicated in regulating the stability of CtBP1 through Lys63-ubiquitination, which improves stability and nuclear retention of CtBP1, thereby facilitating EMT [54]. 

Another typical feature of TMEs is the loss of E-cadherin that is expressed on the surface of cells and is important for cell–cell adhesion, and loss of E-cadherin may result in EMT development and increased cell motility. Hakai, a c-Cbl-like RING E3 ligase, mediates the ubiquitination of E-cadherin in a tyrosine phosphorylation dependent manner. Tyrosine phosphorylation and ubiquitination of E-cadherin is induced by the tyrosine kinase c-Src, which induces endocytosis of E-cadherin [55]. Similarly, RING finger protein 43 (RNF43) also ubiquitinates E-cadherin upon c-Scr activating E-cadherin and promotes EMT progression n lung adenocarcinoma [56]. UBR5, in addition to being involved in the activation of immune cells, is implicated in the stability of E-cadherin as well. Loss of UBR5 results in decreased expression of E-cadherin, which then causes aberrant EMT and reduced tumor metastasis. After ruling out the possibility of UBR5 regulating E-cadherin through promoter hypermethylation, Liao et al. speculated that UBR5 ubiquitinates certain transcriptional repressors of E-cadherin and accordingly found that Snail1 and Twist levels were slightly elevated in tumor cells, although further study is needed to better delineate the molecular mechanism [41]. Novel retinoblastoma E3 ubiquitin ligase (NRBE3), an E3 ligase of the retinoblastoma protein, was also found to negatively regulate E-cadherin expression and is overexpressed in breast cancer [57]. One DUB, USP48, is implicated in the stability of E-cadherin. In response to TNF-α, serine phosphorylation of USP48 by GSK3β increases USP48 activity. Subsequently, USP48 deubiquitinates and stabilizes Lys48-polyubiquitinated TRAF2. TRAF2 is a second messenger adaptor protein in the JNK pathway and negatively regulates the E-cadherin expression [58]. 

### 2.3. The Extracellular Matrix

**Figure 4:**
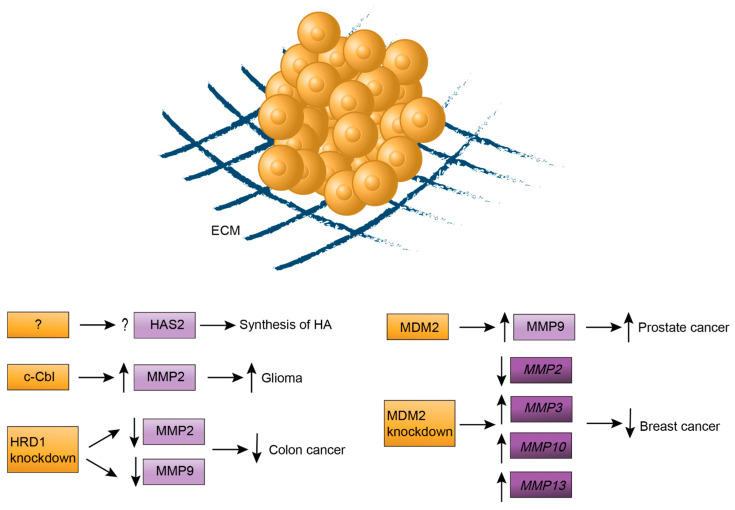
Ubiquitin signaling regulates components of the extracellular matrix (ECM). Hyaluronan (HA) and matrix metalloproteinase 2 (MMP2) are important components of ECM. The enzymes involved in ubiquitination of HAS2 and the effect of ubiquitination on the synthesis of HA are unknown. The E3 ligases c-Cbl, HRD1 and MDM2 regulate MMP protein levels (light purple rectangles) and *MMP* genes expression (dark purple rectangles), which therefore affect cancer development.

The extracellular matrix (ECM) is a complicated macromolecular network composed of various non-cellular components, including collagens, galectins, polysaccharides, glycoproteins and proteoglycans, which are produced by all types of cells in the TME. Various enzymes, such as MMP, growth factors and cytokines, are also present in the ECM. The ECM serves as a structural scaffold for the cells and provides binding sites for soluble factors in the TME [15,16,17]. As expected, ubiquitination influences the presence of components in ECM and in turn regulates tumor development (Figure 4). 

Hyaluronan (HA) is a glycosaminoglycan and an important component of the extracellular matrix. An HA-rich environment can promote tumor progression and metastasis through HA interaction with receptors on cancer cell surfaces, such as CD44. While high-molecular-weight HA is implicated in tumor proliferation, low-molecular-weight HA plays a role in angiogenesis. 

The synthesis and metabolism of HA is controlled by HA synthases (HAS) and three isoforms of HAS have been characterized: HAS1, HAS2 and HAS3 [59]. The activity of HAS2 and synthesis of HA are regulated by ubiquitination and Lys190 of HAS2 was found to be a ubiquitination site [60]. However, the mechanism of how HAS2 is ubiquitinated and the specific effect of ubiquitination on HAS2 are not completely understood.

The RING E3 ligase c-Cbl not only regulates PD-L1 expression but also MMP2 expression [61]. MMP belongs to a family of zinc-dependent endopeptidases and plays a role in ECM degradation, which facilitates tumor metastasis and progression [62]. c-Cbl also upregulates the expression of MMP2, which results in promoted glioma invasion. The possible underlying mechanism is that ubiquitination by c-Cbl may induce the degradation of a protein inhibitor or activation of a positive regulator in the MMP2 production pathway, therefore increased MMP2 production. Further studies are needed to reveal substrates of c-Cbl and how c-Cbl promotes glioma invasion through upregulating MMP2 expression [61]. Furthermore, knockdown of the E3 ligase HRD1 was found to decrease the expression of MMP2 and MMP9 in colon cancer and inhibits the migration and invasion of cancer cells, but the underlying molecular mechanism is unclear [63]. MDM2 is an E3 ligase best-known as a negative regulator of p53 through ubiquitination-mediated proteasomal degradation and transcriptional regulation. MDM2 is positively related to MMP9 expression and secretion in prostate cancer cells, however, in a p53-independent mechanism [64]. In addition, knockdown of MDM2 results in a reduction of *MMP2* gene expression and increase in *MMP3*, *MMP10* and *MMP13* gene expression and decreased growth and migration of breast cancer cells, and the mechanism may involve the interplay between MDM2 and prostate-specific membrane antigen (PSMA) [65]. 

### 2.4. Ubiquitination Regulation in Hypoxia

**Figure 5:**
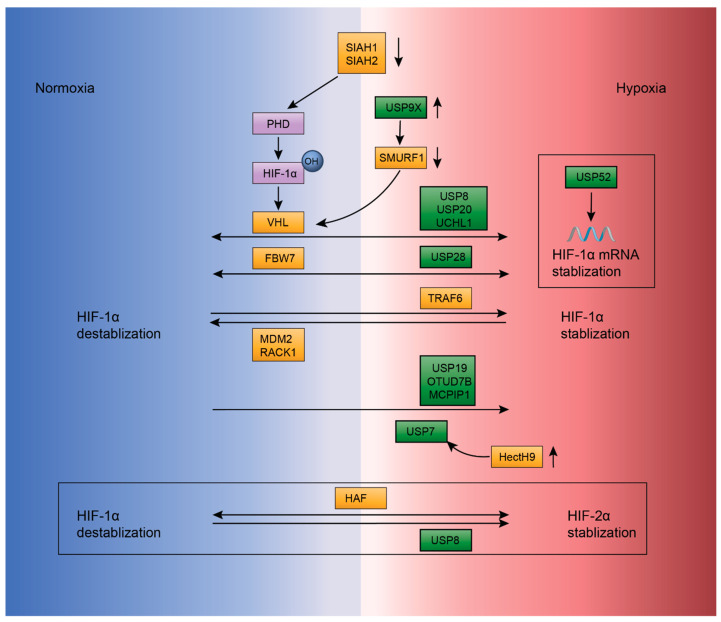
E3s and DUBs involved in modulating the abundance of hypoxia-inducible transcription factor (HIF). HIF (violet rectangles) is the key regulator of the hypoxic response in cells and is composed of the well-characterized HIF-1α and HIF-2α subunits. von Hippel-Lindau (VHL) regulates the ubiquitination-mediated degradation of HIF-1α, which requires hydroxylation of HIF-1α by prolyl hydroxylase (PHD) (violet rectangles) to occur. Two other E3s (yellow rectangles), SIAH1 and SIAH2, are implicated in the downregulation of PHD. VHL is downregulated by an E3 ligase SMURF1 that is stabilized by a DUB (green rectangles) USP9X. USP8, USP20 and UCHL1 counteract the VHL-mediated ubiquitination of HIF-1α. FBW7 ubiquitinates HIF-1α, which is antagonized by USP28. TRAF6, MDM2 and RACK1 are also involved in regulating HIF-1α. USP7, USP19, OTUD7B and MCPIP1 stabilize HIF-1α, and HectH9 is required for USP7 activity. HAF and USP8 mediate HIF-1α and HIF-2α stabilization. USP52 regulates the stabilization of HIF-1α mRNA.

Hypoxia is a distinctive feature of TMEs that results from the high oxygen demand necessary for tumor proliferation. In response to the decreased oxygen in the tumor microenvironment, several cellular pathways are activated in cancer cells and one of the key regulators in the activation process is the hypoxia-inducible transcription factor (HIF). Under hypoxic conditions, HIF exists as a heterodimer composed of the oxygen-regulated α subunit and the oxygen-independent β subunit. HIF-α subunits include HIF-1α, HIF-2α and HIF-3α, of which HIF-1α, HIF-2α are well-characterized in humans [12]. Post-translational modifications play an important role in the abundance and stability of HIF-α. Ubiquitination of HIF-α has been extensively studied and many E3 ligases and DUBs are implicated in regulating HIF-1α protein levels, as discussed below (Figure 5).

Several E3 ligases mediate the ubiquitination of HIF-1α and target it for proteasome degradation. In normoxia, a von Hippel-Lindau (VHL) E3 ligase is responsible for the ubiquitination-mediated degradation of HIF-1α. The interaction between VHL and HIF-1α depends on the hydroxylation of two HIF-1α prolines, Pro402 and Pro564, in the oxygen-dependent degradation (ODD) domain by prolyl hydroxylases (PHDs) [66,67]. SIAH1 and SIAH2, two RING E3 ligases, regulate the ubiquitination and degradation of PHDs and therefore regulate the protein level of HIF-1α indirectly [68,69]. Hydroxylation of HIF-1α requires oxygen, thus HIF-1α cannot be hydroxylated in hypoxia. Other E3 ligases can ubiquitinate HIF-1α in an oxygen-independent manner, such as F-box and WD repeat domain-containing 7 (FBW7), an SCF E3 ligase that ubiquitinates HIF-1α and promotes its degradation, a process in which GSK3β-mediated phosphorylation of HIF-1α is required [67,69]. Hypoxia-associated factor (HAF), another E3 ligase, catalyzes HIF-1α ubiquitination and HIF-2α transcriptional activation, both of which depend on activation of the NF-κB pathway [69,70,71]. Another RING E3 ligase TRAF6 can induce the Lys63-linked polyubiquitination of HIF-1α. However, instead of targeting HIF-1α for degradation, ubiquitination by TRAF6 increases the abundance of HIF-1α [69,72]. MDM2, an important negative regulator of the well-known tumor suppressor p53, acts as a E3 ligase and catalyzes the ubiquitination and degradation of HIF-1α in a p53-dependent manner. HIF-1α also plays a role in the degradation of p53 by hindering the MDM-mediated ubiquitination of p53 [69,73]. In addition to E3 ligases, receptor of activated protein C kinase 1 (RACK1), an anchoring protein for activated protein kinase C, mediates the ubiquitination and degradation of HIF-1α as well by competing with heat shock protein 90 (HSP90) for binding to HIF-1α, which tends to stabilize HIF-1α [69,74].

On the other hand, HIF-α is stabilized by a variety of DUBs. USP8 [75], USP20 [76] and ubiquitin carboxyl-terminal hydrolase L1 (UCHL1) [69,77] counteract the VHL-mediated ubiquitination of HIF-1α. USP8 binds to the PERN-ARNT-SIM (PAS) domain of HIF-1α and HIF-2α and protect them from VHL-mediated ubiquitination [75]. USP20, also known as VHL-interacting deubiquitinating enzyme 2 (VDU2), was the first DUB reported to reverse the VHL-mediated ubiquitination of HIF-1α [76]. Moreover, VHL can ubiquitinate USP20 in return and target it for degradation [78]. UCHL1 interacts directly with HIF-1α and inhibits binding with VHL and HIF-1α, thereby protecting HIF-1α from degradation [77]. In addition, USP28 antagonizes FBW7-mediated ubiquitination and stabilizes HIF-1α [69,79]. USP7, also known as HAUSP (herpesvirus-associated ubiquitin-specific protease), deubiquitinates HIF-1α and stabilizes it. The activity of USP7 relies on Lys63-linked polyubiquitination by the E3 ligase HectH9 (also known as HUWE1), which is induced by hypoxia [69,80]. USP19 protects HIF-1α from ubiquitination-mediated degradation through interacting directly with the PAS domain of HIF-1α independently of its catalytic activity [69,81]. OTUD7B, also known as Cezanne, deubiquitinates Lys11-linked Ub chains and rescues HIF-1α from lysosomal degradation. Loss of OTUD7B leads to degradation of HIF-1α in a VHL dependent manner independent of PHD-mediated prolyl hydroxylation [69,82]. Monocyte chemoattractant protein-induced protein 1 (MCPIP1), which can function as both an RNase and a DUB, deubiquitinates and stabilizes HIF-1α [69,83]. Finally, DUBs can also influence the stability of HIF-1α indirectly. USP52 increases HIF-1α protein levels through stabilizing HIF-1α mRNA instead of affecting HIF-1α ubiquitination [69,84]. USP9X stabilizes SMURF1, an E3 ligase of VHL, which consequently destabilizes VHL and activate HIF pathway [85].

## 3. Modulators Targeting E3s and DUBs in Tumor Microenvironment

### 3.1. Small Molecule Inhibitors Targeting E3s and DUBs

Ubiquitination is widely implicated in the stability of various cellular and non-cellular components in the TME, thereby affecting tumor progression, metastasis and angiogenesis. Targeting TME-related E3s and DUBs provides potential therapeutic approaches and some small molecule inhibitors have been developed for this purpose.

**Figure 6:**
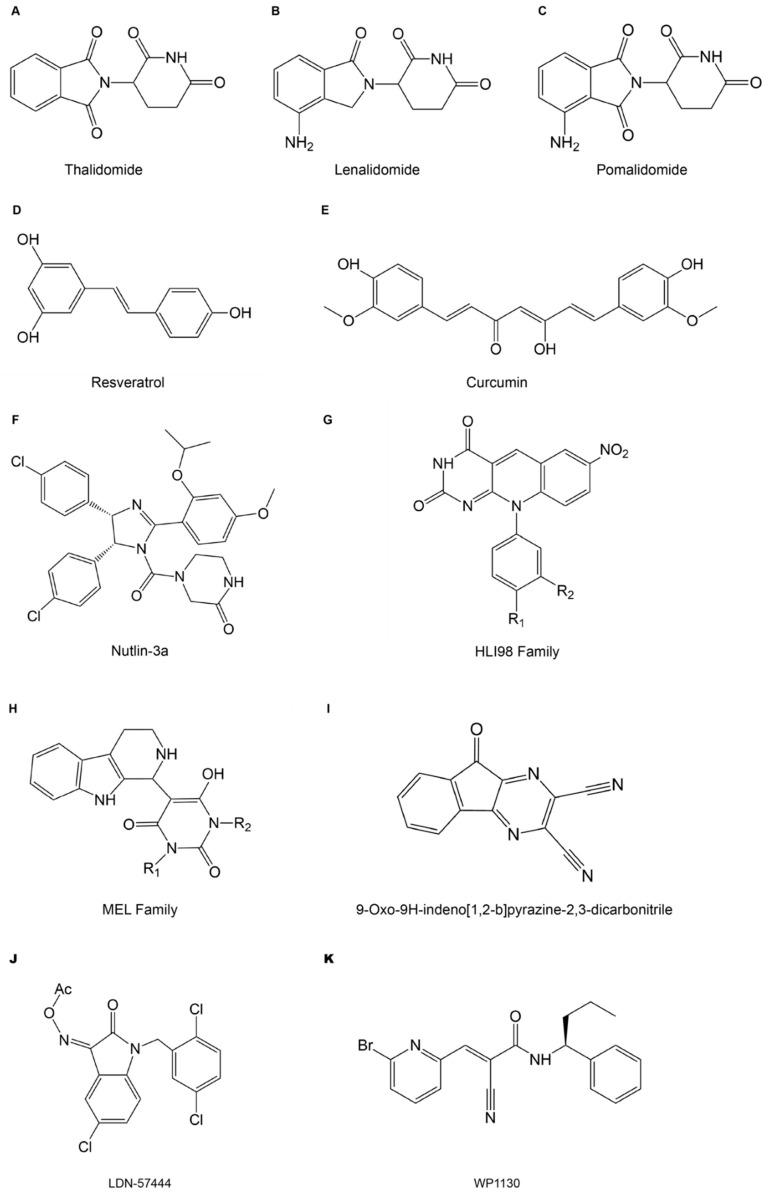
Chemical structures of small molecule inhibitors. (**A**–**C**) Immunomodulatory drugs (IMiDs): thalidomide (**A**) and its derivatives lenalidomide (**B**) and pomalidomide (**C**). (**D**–**E**) PD-L1 modulatory drugs: resveratrol (**D**) and curcumin (**E**). (**F**–**H**) MDM2 inhibitors: nutlin-3A (**F**), HLI98 family (**G**) and MEL family compounds (**H**). (**I**) USP8 inhibitor: 9-oxo-9*H*-indeno [1,2-b]pyrazine-2,3-dicarbonitrile. (**J**) UCHL1 inhibitor: LDN-57444. (**K**) USP9X inhibitor: WP1130.

Small molecule inhibitors have been developed to target immune cell associated E3s and DUBs. Immunomodulatory drugs (IMiDs), including thalidomide and its derivatives lenalidomide and pomalidomide (Figure 6A–C), are used to treat multiple lymphoma and myeloma, such as B-cell non-Hodgkin lymphoma, and have been used clinically. The E3 ligase cereblon (CRBN) was found to be one molecular target of IMiDs. The substrates of CRBN include two transcriptional regulators, Ikaros and Aiolos, which are responsible for the maturation of B cells [86]. Lenalidomide inhibits autoubiquitination of CRBN and promotes its enzymatic activity, which results in increased ubiquitination and degradation of Ikaros and Aiolos. Lenalidomide also has anti-tumor effects, including G0/G1 arrest, reduced number of malignant B cells, decreased inflammatory cytokines, increased numbers of T cells and NK cells, and increased levels of anti-inflammatory cytokines [87].

Modulators targeting E3s or DUBs involved in regulating ubiquitination of PD-L1 have been developed as well. Resveratrol (Figure 6D) has anti-tumor effects in breast cancers through blocking N-glycosylation and promoting dimerization of PD-L1, thus reducing N-glycosylation-mediated interaction with PD-1 [18,88]. Moreover, resveratrol also hinders translocation of PD-L1 to the cell membrane, consequently reducing its interaction with PD-1 and inducing T cell activity [88]. Resveratrol induces the expression of the E3 ligase β-TrCP, which catalyzes ubiquitination of non-glycosylated PD-L1 and thus causes reduced PD-L1 expression and enhanced anti-tumor T cells immunity in breast cancer [18,25]. Another inhibitor, curcumin (Figure 6E), inhibits a JAMM DUB CSN5 that deubiquitinates and stabilizes PD-L1, thereby destabilizing PD-L1 in various cancers [18,30]. Combined treatment using inhibitors that target cytotoxic T-lymphocyte-associated protein 4 (CTLA-4) together with PD-1 has achieved significant clinical success in treating various cancers, especially melanoma, and resulted in higher survival rates of patients [89]. Treatment with curcumin also increases the susceptibility of cancer cells to anti-CTLA4 therapy and inhibits tumor growth [30].

During hypoxia, HIF adapts cancer cells to the hypoxic environment and several inhibitors for hypoxia related E3s and DUBs have been developed. MDM2 and its interaction with p53 are not only involved in the ubiquitination of HIF-1α but also other aspects related to tumor progression. Inhibitors targeting MDM2 have been developed with the aim of interfering with its interaction with p53 to stabilize p53, such as Nutlin-3a [90], HLI98 [91], MEL23, MEL24 [92] and others (Figure 6F–H). Nutlin-3a has been reported to inhibit MDM2 to reduce angiogenesis and metastasis of neuroblastoma in mice [93]. Nutlin-3a also shows anti-tumor effects in multiple types of cancer [94], but whether Nutlin-3a can modulate HIF-1α is not clear so far. HLI98 [91] and MEL family compounds [92] can also inhibit MDM2 E3 ligases to stabilize the tumor suppressor p53. However, their effects on tumor progression still need further investigation, not to mention their potential effects on HIF-1α. Active inhibitors of USP8, 9-oxo-9*H*-indeno [1,2-b]pyrazine-2,3-dicarbonitrile (Figure 6I) and its analogues have been identified, but the underlying mechanism and its effects on cancer cells have yet to be investigated [95]. The inhibitor of UCHL1, LDN-57444 (Figure 6J), blocks the UCHL1-HIF1 axis and therefore inhibits HIF-1 activity as UCHL1 deubiquitinate and stabilizes HIF-1α. UCHL1 promotes distant tumor metastasis and treatment with LDN-57444 inhibits pulmonary metastasis effectively in mice [77]. In addition to cancers, LDN-57444 also inhibits development of atrial fibrillation in mouse models through blocking multiple signaling pathways including UCHL1-HIF1 pathway [96]. Finally, USP9X plays a role in regulating HIF indirectly through regulating the E3 ligase of VHL. An inhibitor of USP9X, WP1130 (Figure 6K), was reported. WP1130 induces tumor cell apoptosis through inhibiting USP9X, therefore decreasing the protein level of one other substrate of USP9X, antiapoptotic protein MCL-1 [97]. WP1130 also increases cisplatin sensitivity of estrogen receptor-negative breast cancer in a USP9X-dependent manner [98]. It also confers doxorubicin sensitivity of hepatocellular carcinoma through USP9X-dependent degradation of p53 [99]. However, how exactly the inhibition of USP9X by WP1130 affects HIF signaling is poorly understood. In addition to USP9X, WP1130 can also target other DUBs including DUB3. As mentioned above, DUB3 stabilizes Snail1, a key transcriptional factor in EMT. WP1130 inhibits DUB3 and promotes Snail1 degradation. Treatment with WP1130 reduces tumor cell migration, invasion and tumor mammosphere formation in mice with breast cancer [51]. USP24 was also found to be one of targets of WP1130 T-cell acute lymphoblastic leukemia (T-ALL). Treatment with WP1130 induces apoptosis of T-ALL cells through inhibiting USP24 and therefore its substrate MCL-1. USP24 induces IL-6 in TME through deubiquitinating p300 and β-TrCP, and whether WP1130 inhibiting USP24 affects IL-6 in TME needs more investigation [100].

### 3.2. Ubiquitin Variants as Modulators of E3 Ligases and Deubiquitinases

**Figure 7:**
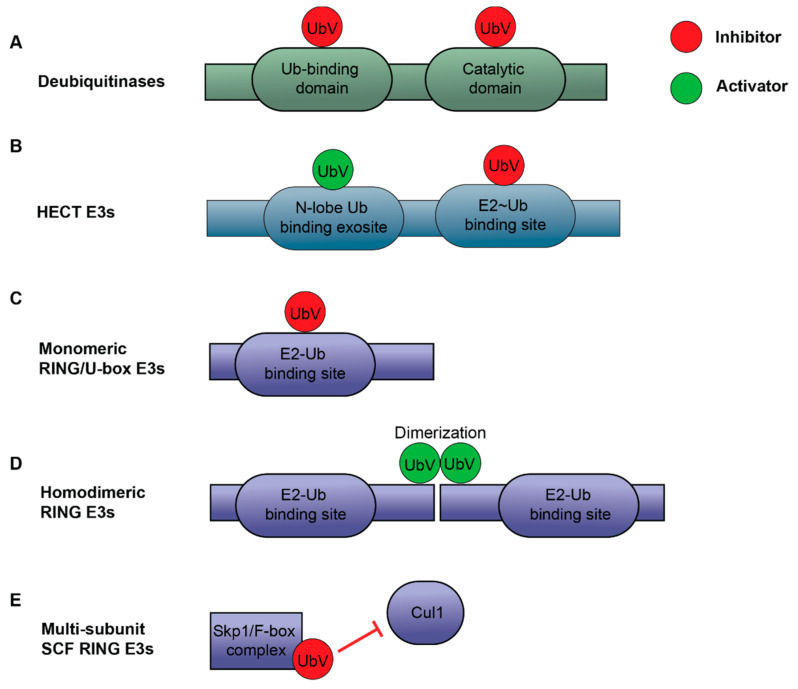
Ubiquitin variants (UbVs) as modulators of the ubiquitin proteasome system (UPS) components. (**A**) UbVs can bind to either the catalytic domain or the ubiquitin (Ub) binding domain, thereby inhibiting the parent protein’s function, as seen in the USP subfamily of DUBs. (**B**) UbVs are either activating or inhibitory for HECT-family E3 ligases depending on the binding site on the HECT domain. (**C**–**E**) UbVs can act as an inhibitor or activator for RING/U-box E3 ligases. (**C**) As an inhibitor for monomeric RING/U-box E3 ligases, UbV prevents E2~Ub binding. (**D**) For homodimeric RING E3 ligases (e.g., XIAP), UbV can dimerize to engage binding with homodimeric RING domain to stimulate E3 activity (middle). (**E**) For multi-subunit SCF-family RING E3 ligases, UbV disrupts the complex formation to inhibit catalytic functions of E3 ligases.

To date, the development of small molecule inhibitors demonstrated the feasibility of targeting E3 ligases and DUBs in cancer therapy. However, many challenges and drawbacks remain, including lack of specificity and mild potency, which compromise their efficacy in cells and animal models [101]. To bypass such disadvantages, researchers have devised a protein engineering strategy to develop inhibitors utilizing ubiquitin due to its low affinity but high specificity of binding to E3s and DUBs [102]. Ubiquitin is a small but highly stable protein [103], thus representing a unique candidate subjected to structure-based combinatorial engineering methods. Indeed, the Sidhu group has constructed a diverse phage-displayed library containing > 10 billion ubiquitin variants (UbVs) by introducing mutations to the ubiquitin surface mediating numerous interactions with other proteins in the ubiquitin signaling [102]. Employing phage display-based selection methods, UbV inhibitors and activators with increased affinity and high specificity for many ubiquitin proteasome system (UPS) components are generated [102,104,105,106,107,108,109,110,111,112,113]. It is therefore conceivable that this technology can be applied to develop potent modulators of E3s and DUBs related to TME components as novel therapeutics for cancer treatment. Below, we briefly introduce several examples of how UbVs can be developed to modulate the catalytic activity of DUBs and E3 ligases (Figure 7).

#### 3.2.1. UbV Inhibitors for DUBs

The first UbV library was developed by structure-based combinatorial engineering of approximately 30 residues on Ub surface that are responsible for interaction with the USP subfamily of DUBs [102] (Figure 7A). Subsequently, billions of UbVs were displayed on M13 phages and binders were selected for various UPS components. For example, this approach has yielded UbVs that bind tightly and selectively to the USP-family DUBs USP8, USP21 or USP2a. Further structural analysis showed that the specificity of binding resulted from interactions mediated by mutated residues in areas that are not conserved in the USP subfamily. In addition, biochemical assays and cellular experiments confirmed the inhibitory activity of these UbVs. Selective binders were also identified for other DUB families, such as OTUB1 of the OTU family and Brcc36-containing isopeptidase complex (BRISC) of the JAMM family [102]. With the exception of a UbV that bound to the non-catalytic ubiquitin binding domain of USP37, all UbVs in this study bound to a distal Ub-binding site to inhibit substrate binding and poly-Ub chain cleavage activity [102]. Furthermore, Leung et al. [114] used combinatorial saturation scanning to determine the effects of all possible mutations across a protein-binding surface and this can be used as an efficient method of assessing each UbV binding surface to develop more inhibitors of other DUB families.

Another group came out with a different approach to design the library of UbVs through diversifying residues of the β1-β2 loop in the Ub core (with computational prediction) to target USP-family DUBs [105]. UbV inhibitors for USP7 (the most potent was named “U7Ub25.2540”) with nanomolar affinity were developed [105]. Similarly, UbVs were generated with high affinity for USP14, which inhibited Ub signaling and cell survival in yeast [106]. The remarkable ability for a relatively small number of residue changes and mild moderations in Ub conformation to increase binding affinity and specificity has implications for utilizing UbVs to understand the details of Ub-USP interactions.

In a follow-up study using the phage library of UbVs bearing surface residue randomization, three UbVs that bound to USP7 (UbV.7.1, 7.2 and 7.3) were produced [108]. In a side-by-side comparison to the USP7 UbVs with core residue diversification [106], UbV.7.2 outperformed U7Ub25.2540 by 10- to 25-fold in various assays and bound to USP7 more specifically while tested against a panel of 23 different DUBs [108]. Furthermore, it was shown that UbV.7.2 inhibited activity of USP7 in vitro and in cells; and conferred chemotherapeutic synergistic effect in combination with cisplatin for increasing cancer cell death and improving cancer treatment [108]. Finally, modelling the crystal structure of UbV.7.2 with the USP7-Ub.wt complex followed by mutation analysis suggested that a newly formed hydrophobic patch and the divergent C-terminal region of UbV.7.2 contributed to the enhanced binding with USP7. More recently, Teyra et al. [107] developed inhibitors for USP15, a DUB with diverse cellular functions and substrates. USP15 contains three domains: a DUSP domain (domain present in Ub-specific proteases), two Ub-like (Ubl) domains and a catalytic domain that has two lobes (D1 and D2) separated by a large insert (CD-insert). In addition to the original UbV library [102], this study hard randomized 10–13 positions in Ub, and the constructed next-generation UbV library contained 2.5 × 10^10^ unique variants [107], which were then screened for binders to various USP15 fragments. They were able to develop specific UbVs that bound to each of the four known structured domains within USP15 (DUSP, Ubl-1, Ubl-2 and D1/D2) [107]. Binders to the catalytic domain, despite inhibiting USP15 function in vitro, required optimization of the UbV binding surface to improve efficiency and inhibit DUB interaction with the substrates SMURF2 and TRIM25. They further generated diUbVs that targeted the DUSP and catalytic domains concurrently, which greatly increased the level of USP15 inhibition and reduced its interaction with substrates in the TGF-β pathway [107]. Finally, UbVs were employed as highly selective and potent inhibitors against pathogenic DUBs of Middle East respiratory syndrome coronavirus (MERS-CoV) and the Crimean-Congo hemorrhagic fever virus (CCHFV) at sub-nanomolar concentrations [109].

Taken together, these studies show that UbVs can inhibit enzyme activity like small molecule inhibitors do while being more advantageous due to its high specificity, which makes it an attractive molecule with therapeutic potential. Below, we present several studies extending the UbV technology to not only inhibit E3 ligases, but also activate their enzymatic activity.

#### 3.2.2. UbV Activators and Inhibitors for E3 Ligases

While developing the strategy for generating inhibitors for DUBs using the phage-displayed UbV library, Ernst et al. [102] discovered several UbVs binding to neural precursor cells induced expression of developmentally down-regulated protein 4 (NEDD4), a HECT-family E3 ligase (Figure 7B). Interestingly, it was observed that the UbV.N.2 bound to the HECT domain of NEDD4 and enhanced auto-ubiquitination of NEDD4 in vitro and ubiquitination of the substrate transcription factor Ying-Yang1 (YY1) in cells [102]. Subsequently, we targeted HECT domains of 19 human HECT E3 ligases and the yeast homolog of human NEDD4 and found that while some UbVs inhibited ubiquitination, there were also activators that positively modulated ubiquitin transfer from E2 conjugating enzyme and substrate polyubiquitination [104]. Further structural analysis showed that the inhibitors acted by blocking the E2 binding site and preventing transfer of Ub to the E3 ligase, not by blocking the catalytic site as expected, while the activators bind to a N-lobe exosite [104] that was previously found to interact with Ub [115]. The ability of UbVs to engage different allosteric sites and elicit variable effects on enzyme function further increases their attractiveness as therapeutic agents.

Other families of E3 ligases that are not known to interact directly with Ub have also been targeted for UbV binding and modulation (Figure 7C–E). For example, Gabrielsen et al. [110]. targeted the RING/U-box E3 ligases UBE4B, CBL and XIAP. The UbVs generated in this study had either inhibitory or activating effects. The inhibitory UbV.E4B bound to the U-box domain of UBE4B while UbV.pCBL bound to CBL phosphorylated at Tyr371 and prevented E2 binding, thereby inhibiting Ub transfer [110] (Figure 7C). Conversely, UbV.XR binds to XIAP and enhances its ligase activity by forming a dimer, thereby likely stabilizes dimerization of XIAP and leading to its activation [110] (Figure 7D). Multi-subunit SCF E3 ligases of the RING family have also been studied for UbV binding [111]. UbVs developed in this study prevent Cul1 from binding to the interface of Skp1 and F-box (Figure 7E) and were shown to inhibit ligase activity in vivo. With further modifications, they were able to generate inhibitors that targeted a broad spectrum of SCF ligases with high specificity even between highly similar homologs [111], which is a promising characteristic for developing therapeutics.

APC/C is a multimeric E3 cullin-RING ligase complex, the catalytic core of which contains the cullin subunit APC2, RING protein APC11, adaptor protein APC10 and substrate binding subunits (CDC20 or CDH1) [116]. Brown et al. [113] generated a UbV that binds to the APC11 subunit at the binding surface of a substrate-linked Ub and inhibits Ub chain elongation and multiubiquitination. This UbV has been used to facilitate the understanding of the complex mechanisms of APC/C for multiubiquitination and elongation processes. More recently, Watson et al. [112] identified a UbV that competes with E2 binding, thereby attenuating ubiquitination by hijacking APC2 in its winged-helix B domain.

Collectively, these studies show that highly specific UbVs can be developed for a wide range of protein families in the ubiquitin proteasome system and can act as both inhibitors and activators, which make them more attractive than current small molecule inhibitors for developing therapeutics that target E3s and DUBs in TME. However, while inhibition can be effective, there is an alternative approach involving targeting proteins for degradation rather than simply inhibiting them, which is worth exploring due to the enhanced effectiveness as discussed below.

### 3.3. PROTAC

**Figure 8:**
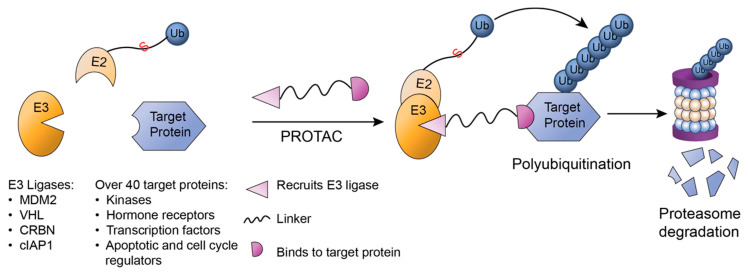
Proteolysis targeting chimeras (PROTACs). PROTAC links E3 ligases to target proteins, facilitating ubiquitination and eventual degradation. A PROTAC molecule has three subunits: one that binds to target protein, one that recruit a E3 ligase, and finally a linker connecting both parts. PROTAC induces the formation of a ternary complex, in which an E3 ligase bound to E2~Ub ubiquitinates the target protein and leads to subsequent proteasomal degradation.

Proteolysis targeting chimeras (PROTACs) are molecules that can recruit the cellular UPS to degrade proteins [117] (Figure 8). The rationale for PROTACs is that bringing the E3 ligase in the vicinity of the protein of interest will trigger ubiquitination by the E3 ligase and subsequent proteasomal degradation even for proteins that are not substrates of the specific E3 ligase [118]. A PROTAC consists of three component: one that binds to a target protein, a ligand that recruits the degradation unit E3 ubiquitin ligase and a linker region connecting these two [119]. The first PROTAC, Protac-1, was peptide-based, targeting methionine aminopeptidase-2 (MetAP-2) for ubiquitination and degradation through recruitment of an SCF complex [120]. Although the effectiveness of Protac-1 was limited due to its large size and limited cell permeability, further efforts have been made to optimize PROTACs by using small molecules known to bind to E3 ligases, such as MDM2, VHL, CRBN and cellular inhibitor of apoptosis protein 1 (cIAP1) [121,122,123,124,125,126]. These studies have established PROTACs as a promising technology for drug discovery and studying protein functions with more than 40 targets degraded to date [127]. The IMiD thalidomide and its derivatives lenalidomide and pomalidomide were shown to bind to the Cul4–Rbx1–DDB1–CRBN E3 ubiquitin ligase complex [128,129] and their anticancer effects are due to CRBN inducing degradation of the IKAROS family transcription factors IKZF1 and IKZF3 [128]. Similarly, an androgen receptor was targeted for degradation by a non-steroidal androgen receptor ligand (SARM) and the MDM2 ligand known as Nutlin, which were connected by a PEG-based linker [121].

PROTACs are attractive for cancer therapy also because they have been exploited to target proteins considered ‘undruggable’, such as kinases and the bromodomain and extra terminal containing (BET) family of proteins [130]. The PROTAC ARV-825, developed due to the limited effectiveness of BET inhibitors (BETi), targets the bromodomain-containing 4 (BRD4) region in BET proteins for CRBN-mediated proteasomal degradation [131]. This PROTAC was more effective at blocking BETP transcriptional regulation of c-Myc and other oncoproteins in myeloproliferative neoplasms than the BETi [131]. Recently, it has been shown ARV-825 also plays a major role in depleting leukemia stem cells by affecting their microenvironment [132]. ARV-825 resulted in reduced BETP-dependent transcription of chemokine receptors, indicated by downregulation of surface CXCR4 and CD44 in leukemia stem cell populations, thereby reducing CD34+CD38– putative leukemia progenitor cell populations without affecting healthy BM–derived progenitor cells [132]. These results were observed in acute myeloid leukemia (AML) cells and sustained in mouse models with patient-derived xenografts [132], which indicate ARV-825′s potential for targeting tumor microenvironments. A major advantage of PROTACs is their mode of action, which does not necessitate binding to the enzymatic site of the target protein, leading to effectiveness even in low concentrations [118]. Additionally, the resistance mechanisms that can be developed over time due to use of inhibitors, such as overexpression of target proteins to outcompete the inhibitor or mutations to prevent proper binding, can be avoided through the use of degraders like PROTACs [130]. In summary, there is great potential for PROTACs to be developed specifically to target TMEs with more effectiveness than other approaches.

## 4. Conclusions

Ubiquitination plays an important role in regulating the activity and stability of both cellular and non-cellular components of the TME. Several E3 ligases and DUBs have been found to regulate the activity of immune cells through modulating the stability of PD-L1, TAM polarization, secretion of cytokines and the activation of immune cells. They are also involved in reprogramming of metabolic pathways, secretion of soluble factors and the EMT process of CAF. Ubiquitination is implicated in the formation of HA and expression of MMP2, which are essential components of the ECM and, in response to hypoxia, E3 ligases and DUBs modulate HIF-1α. Overall, TME-related ubiquitination influences immune responses, tumor progression and metastasis in both direct and indirect ways.

Both cellular and non-cellular components in the TME contribute to tumor development and provide potential targets for cancer treatment. Indeed, in the last decade quite a few of modulators have been developed to target the ubiquitin signaling cascade in the TME. Going forward, we believe that the two emerging technology introduced above can be explored for developing innovative cancer therapeutics.

The UbV platform was enabled by generating a diverse phage-displayed library containing billions of variants with mutated surface residues of Ub, followed by selecting UbV binders to UPS components through modulating the Ub interaction interface. The resulted UbVs are functional inhibitors or activators with increased affinity and high specificity. Importantly, UbVs exposed allosteric modulating surfaces on E3 ligases and DUBs that small molecule inhibitors failed to reveal. Moreover, structural studies of the UbV-protein complex provided critical information for further improvement of these synthetic molecules and design or screening of chemical compound inhibitors. Taken together, the UbVs can be developed to modulate E3 ligases and DUBs involved in TME to serve as intracellular probes for target validation, identification of new substrates and proteomics studies. The recombinant nature of UbVs is also facile for genetic manipulation and direct delivery. Last but not least, UbVs can be integrated into drug development by adopting small molecule displacement screens [133], where compounds that mimic the UbV-binding mode can be identified and act as potential modulators for E3 ligases and DUBs in TME.

Due to its general applicability in targeting “undruggable” proteins, we envision that PROTACs will be broadly employed in the next decade to modulate TME by engaging the catalytic activity of E3 ligases to the proximity of proteins of interest. On the other side of the spectrum, targeted deubiquitination [134] is on the horizon for protein stabilization and cell signaling manipulation. Finally, UbV and its small molecule mimetics can potentially be used to mediate target ubiquitination or deubiquitination as well due to its highly specific E3/DUB binding.

## Data Availability

Not applicable.

## Abbreviations

HECT	Homologous to the E6AP Carboxyl Terminus
RING	Really Interesting New Gene
RBR	RING-Between-RING
DUB	Deubiquitinase
USP	Ubiquitin-Specific Protease
UCH	Ubiquitin C-terminal Hydrolase
OTU	Ovarian Tumor Protease
MJD	Machado-Joseph Disease Protease
JAMM	JAB1/MPR/Mov34 Metalloprotease
MINDY	Motif Interacting with Ubiquitin-Containing Novel DUB Family
TME	Tumor Microenvironment
TAM	Tumor-Associated Macrophage
MDSC	Myeloid Suppressive Cell
DC	Dendritic Cell
NK	Natural Killer
CD	Cluster of Differentiation
Th	T Helper Cell
Treg	Regulatory T Cell
PD-L1	Programmed Death-Ligand 1
PD	Programmed Cell Death protein 1
CHIP	Carboxy Terminus of Hsc70 Interacting Protein
STUB1	STIP1 Homology and U-Box Containing Protein 1
β-TrCP	Β-Transducin Repeat-Containing
CRL	Cullin-RING E3 Ligase
SCF	Skp1-Cullin1-F-Box Protein
GSK3β	Glycogen Synthase Kinase 3β
FBXO	F-Box Only Protein
HRD1	HMG-CoA Reductase Degradation Protein 1
ERAD	Endoplasmic Reticulum Associated Protein Degradation
POZ	Pox Virus and Zinc Finger Protein
SPOP	Speckle-Type POZ Protein
APC/C	Anaphase-Promoting Complex/Cyclosome
CDK	Cyclin Dependent Kinase
c-Cbl	Casitas B-Cell Lymphoma
Cbl-b	Casitas B-Lineage Lymphoma Proto-Oncogene-B
STAT	Signal Transducer and Activator of Transcription Protein
ERK	Extracellular Signal Regulated Kinase
CSN5	COP9 Signalosome Complex Subunit 5
NF-κB	Nuclear Factor-κB
TNF	Tumor Necrosis Factor
CKLF	Chemokine-Like Factor
CMTM6	CKLF-Like Marvel Transmembrane Domain Containing Family Member 6
TAM	Tumor-Associated Macrophage
IL	Interleukin
PGE2	Prostaglandin 2
TGF	Transforming Growth Factor
SIAH	Seven in Absentia Homologue
NRF1	Nuclear Respiratory Factor 1
Trim24	Tripartite Motif-Containing Protein 24
CBP	CREB-Binding Protein
Stat6	Signal Transducer and Activator of Transcription 6
IκB	Inhibitor of NF-κB
DNMT1	DNA Methyltransferase 1
WDR4	WD Repeat 4
PML	Promyelocytic Leukemia
UBR5	Ubiquitin Protein Ligase E3 Component N-Recognin 5
CAF	Cancer-Associated Fibroblasts
EndMT	Endothelial-Mesenchymal-Transition
EMT	Epithelial-Mesenchymal-Transition
MMP	Matrix Metalloproteinase
ATF4	Activating Transcription Factor 4
TRAF	Tumor Necrosis Factor Receptor-Associated Factor
sICAM1	Soluble Intercellular Adhesion Molecule 1
NOX	NADPH Oxidase
ROS	Reactive Oxygen Species
FBXW	The F-box and WD Repeat Domain Containing Protein
FBXL	F-box and Leucine-rich Repeat Protein
TNFAIP3	Tumor Necrosis Factor α-induced Protein 3
CtBP1	C-Terminal Binding Protein 1
RNF43	RING Finger Protein 43
NRBE3	Novel Retinoblastoma E3 Ubiquitin Ligase
JNK	c-Jun N-terminal Kinase
ECM	Extracellular Matrix
HA	Hyaluronan
HAS	HA Synthase
PSMA	Prostate Specific Membrane Antigen
HIF	Hypoxia Inducible Transcription Factor
VHL	von Hippel-Lindau E3 ligase
ODD	Oxygen Dependent Degradation
PHD	Prolyl Hydroxylase
FBW	F-box and WD Repeat Domain-containing
HAF	Hypoxia Associated Factor
MDM2	Mouse Double Minute 2 Homolog
RACK1	Receptor of Activated Protein C Kinase
HSP	Heat Shock Protein
UCHL1	Ubiquitin Carboxyl-terminal Hydrolase L1
PAS	PERN-ARNT-SIM Domain
VDU2	VHL-interacting Deubiquitinating Enzyme 2
HAUSP	Herpesvirus Associated Ubiquitin Specific Protease
HectH9	Homologous to E6AP Carboxyl Terminus Homologous Protein 9
HUWE1	HECT, UBA and WWE Domain Containing E3 Ubiquitin Protein Ligase 1
MCPIP1	Monocyte Chemoattractant Protein Induced Protein 1
SMURF	SMAD Specific E3 Ubiquitin Protein Ligase
IMiD	Immunomodulatory Drug
CRBN	Cereblon
CTLA-4	Cytotoxic T Lymphocyte Associated Protein 4
HLI98	HDM2(also known as MDM2) ligase inhibitor 98
MEL23/24	Mdm2 E3 Ligase Inhibitors 23/24
MCL	Myeloid Cell Leukemia
T-ALL	T-cell Acute Lymphoblastic Leukemia
UbV	Ubiquitin Variant
UPS	Ubiquitin Proteasome System
BRISC	Brcc36-containing isopeptidase complex
DUSP	Domain Present in Ub Specific Proteases
MERS-CoV	Middle East Respiratory Syndrome Coronavirus
CCHFV	Crimean-Congo Hemorrhagic Fever Virus
NEDD4	Neural Precursor Cells Induced Expression of Developmentally Down-regulated Protein 4
YY1	Ying-Yang1
UBE4B	Ubiquitination Factor E4B
XIAP	X-linked Inhibitor of Apoptosis
CDC20	Cell Division Cycle protein 20
CDH1	CDC20 Homolog-1
PROTAC	Proteolysis Targeting Chimeras
MetAP-2	Methionine Aminopeptidase-2
cIAP1	Cellular Inhibitor of Apoptosis Protein 1
IKZF1/3	Ikaros Family Zinc Finger Protein 1/3
SARM	Steroidal Androgen Receptor Ligand
BET	Bromodomain and Extra Terminal Domain
BETi	BET inhibitors
BETP	BET-Containing Protein
BRD4	Bromodomain-Containing 4
CXCR4	C-X-C chemokine receptor type 4
AML	Acute Myeloid Leukemia
